# Transvenous Lead Extraction during Cardiac Implantable Device Upgrade: Results from the Multicenter Swiss Lead Extraction Registry

**DOI:** 10.3390/jcm12165175

**Published:** 2023-08-08

**Authors:** Andreas Haeberlin, Fabian Noti, Alexander Breitenstein, Angelo Auricchio, Tobias Reichlin, Giulio Conte, Catherine Klersy, Moreno Curti, Etienne Pruvot, Giulia Domenichini, Beat Schaer, Michael Kühne, Michal Gruszczynski, Haran Burri, Richard Kobza, Christian Grebmer, François D. Regoli

**Affiliations:** 1 Department of Cardiology, Inselspital, Bern University Hospital, University of Bern, 3015 Bern, Switzerland; 2 University Hospital Zurich, University Heart Center Zurich, 8091 Zurich, Switzerland; 3 Cardiology Department, Cardiocentro Ticino Institute, 6900 Lugano, Switzerland; 4 Biostatistics and Clinical Trial Center, Fondazione IRCCS San Matteo di Pavia, 27100 Pavia, Italy; 5 Department of Cardiology, CHUV, 1011 Lausanne, Switzerland; 6 Department of Cardiology, University Hospital of Basel, 4002 Basel, Switzerland; 7 Heart Surgery, Stadtspital Triemli, 8091 Zurich, Switzerland; 8 Department of Cardiology, HUG, 1205 Geneva, Switzerland; 9 Department of Cardiology, Luzerner Kantonsspital, 6004 Luzern, Switzerland; 10 Department of Cardiology Service, San Giovanni Hospital, Cardiocentro Ticino Institute, 6500 Bellinzona, Switzerland

**Keywords:** transvenous lead extraction, upgrade procedure, lead extraction indication, lead extraction complications, lead extraction risk factors

## Abstract

Background: Device patients may require upgrade interventions from simpler to more complex cardiac implantable electronic devices. Prior to upgrading interventions, clinicians need to balance the risks and benefits of transvenous lead extraction (TLE), additional lead implantation or lead abandonment. However, evidence on procedural outcomes of TLE at the time of device upgrade is scarce. Methods: This is a post hoc analysis of the investigator-initiated multicenter Swiss TLE registry. The objectives were to assess patient and procedural factors influencing TLE outcomes at the time of device upgrades. Results: 941 patients were included, whereof 83 (8.8%) had TLE due to a device upgrade. Rotational mechanical sheaths were more often used in upgraded patients (59% vs. 42.7%, *p* = 0.015) and total median procedure time was longer in these patients (160 min vs. 105 min, *p* < 0.001). Clinical success rates of upgraded patients compared to those who received TLE due to other reasons were not different (97.6% vs. 93.0%, *p* = 0.569). Moreover, multivariable analysis showed that upgrade procedures were not associated with a greater risk for complications (HR 0.48, 95% confidence interval 0.14–1.57, *p* = 0.224; intraprocedural complication rate of upgraded patients 7.2% vs. 5.5%). Intraprocedural complications of upgraded patients were mostly associated with the implantation and not the extraction procedure (67% vs. 33% of complications). Conclusions: TLE during device upgrade is effective and does not attribute a disproportionate risk to the upgrade procedure.

## 1. Introduction

Implantation numbers of complex cardiac implantable electronic devices, such as cardiac resynchronization therapy (CRT) or conduction system pacing (CSP) devices are on the rise [1,2]. Device upgrade procedures from simpler to more complex systems are associated with a significant risk of complications [3] and the expanded use of such devices at a later disease stage often necessitates advanced lead management decisions. In general, a higher total lead burden may also increase the prevalence of lead–lead interactions, tricuspid valve regurgitation and central venous occlusion syndromes. Thus, clinicians can be confronted with the challenging and controversial choice of lead abandonment in conjunction with additional lead implantation or transvenous lead extraction (TLE) [4]. Within experienced high-volume centers, TLE is a relatively safe procedure that can be performed with high success rates [5,6,7]. Accordingly, current guidelines suggest that TLE may be considered to reduce the total lead burden and maintain venous patency during device upgrade procedures [8]. Moreover, venous occlusion may preclude any device upgrade attempt, necessitating TLE in order to perform the upgrade in the first place [9,10]. Finally, future risks of previously abandoned leads are not negligible; they constitute important additional risk factors in case of a potential future TLE [11].

While some encouraging reports on TLE during upgrade procedures have been published [12,13], general evidence on procedural outcomes of TLE at the time of device upgrade is still scarce. In this nationwide observational registry, we report our experience with TLE during device upgrade procedures.

## 2. Methods

### 2.1. Study Design and Patient Population

The present study represents a post hoc analysis of patients who underwent TLE and were included in the multicenter Swiss TLE registry. This is an investigator-initiated non-sponsored multicenter registry that includes data on TLE procedures performed in eight tertiary Swiss centers from January 2013–December 2021. The regional ethics committee of each participating center approved the study design and protocol (2018-01540; 2018-00253). The investigation conformed with the principles outlined in the Declaration of Helsinki.

### 2.2. Study Objectives

The objectives of this study were to assess patient and procedural factors influencing TLE outcomes at the time of device upgrade.

### 2.3. Definitions

Definitions published in the consensus documents by EHRA and by HRS were used to define procedural approaches, techniques and outcomes [8,14]. In brief, sheaths were classified as mechanical non-powered (polypropylene or similar plastic material), or powered (laser, radio-frequency electrosurgical, or controlled-rotational with threaded tip devices). TLE safety and efficacy were analyzed by considering the rate of procedure-related complications and success/failures (radiological and clinical). A major complication was defined as one related to the procedure that was life-threatening or resulted in death, or any unexpected event that caused persistent or significant disability, or any event that required significant surgical intervention. A radiological failure was defined when more than a 4 cm length of lead was abandoned after a removal attempt, partial success when less than 4 cm of lead remained in the patient’s body and complete success when the lead was completely removed. Clinical failure (considered for each patient) was defined when either a procedure-related major complication or a failure to achieve the clinical outcome for which the TLE was scheduled occurred.

### 2.4. Statistical Analysis

Univariable analysis was applied to both continuous and categorical variables. Continuous variables were reported as mean± standard deviation (SD) or as median and inter-quartile range (IQR). Comparisons between indication groups (upgrades vs. others) were performed using a non-parametric test (Mann–Whitney U test). Categorical variables were reported as percentages. Group comparisons were made using Fisher’s exact test. 

Correlation analysis of TLE interventions per center and resulting complication rates were performed using Spearman’s rank correlation coefficient. Kaplan–Meier curves for the cumulative probability of major complications, TLE-related complications, and deaths at 30 days were plotted and compared between indication groups of patients with the Log-Rank test. A multivariable Cox regression model was used to determine the association between pre-defined clinical variables and any major complication after TLE. The pre-defined variables were age groups (≤69 vs. >69 years), gender, upgrade indication, ELECTRa Registry Outcome Score (EROS) groups 1–3 (low, intermediate, and high risk), LVEF group (≤50% vs. >50%), renal impairment, BMI, systemic infection indication, and dual coil ICD leads. Hazard ratios (HR) and 95% confidence intervals (95%-CI) were calculated.

A two-sided *p*-value of 0.05 was considered statistically significant. Statistical analysis was performed using Stata version 17 (StataCorp, College Station, TX, USA).

## 3. Results

### 3.1. Patient Characteristics and the Increasing Importance of TLE during Upgrade

**Figure 1:**
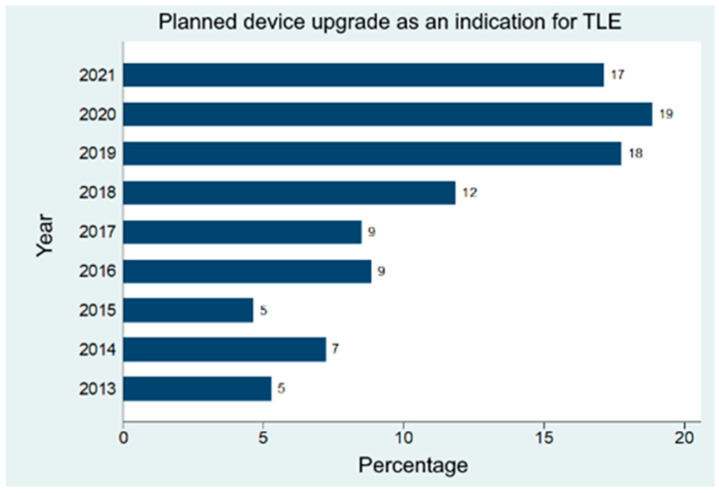
Rate of TLE procedures due to planned device upgrades over the past years.

A total of 941 patients were included in the registry. In 83 cases (8.8%), the reason for TLE was a planned upgrade. Over the past several years, TLE was increasingly more often performed prior to planned device upgrades in order to gain vascular access or reduce the number of indwelling leads (Figure 1). 

Patient baseline characteristics for upgraded patients and patients that underwent TLE due to other reasons are shown in Table 1 (missing data for most variables were <10%). Upgraded patients were more often male, had a lower left ventricular ejection fraction, and received more often oral anticoagulants. 

### 3.2. Risk Factors for TLE during Device Upgrade

**Figure 2:**
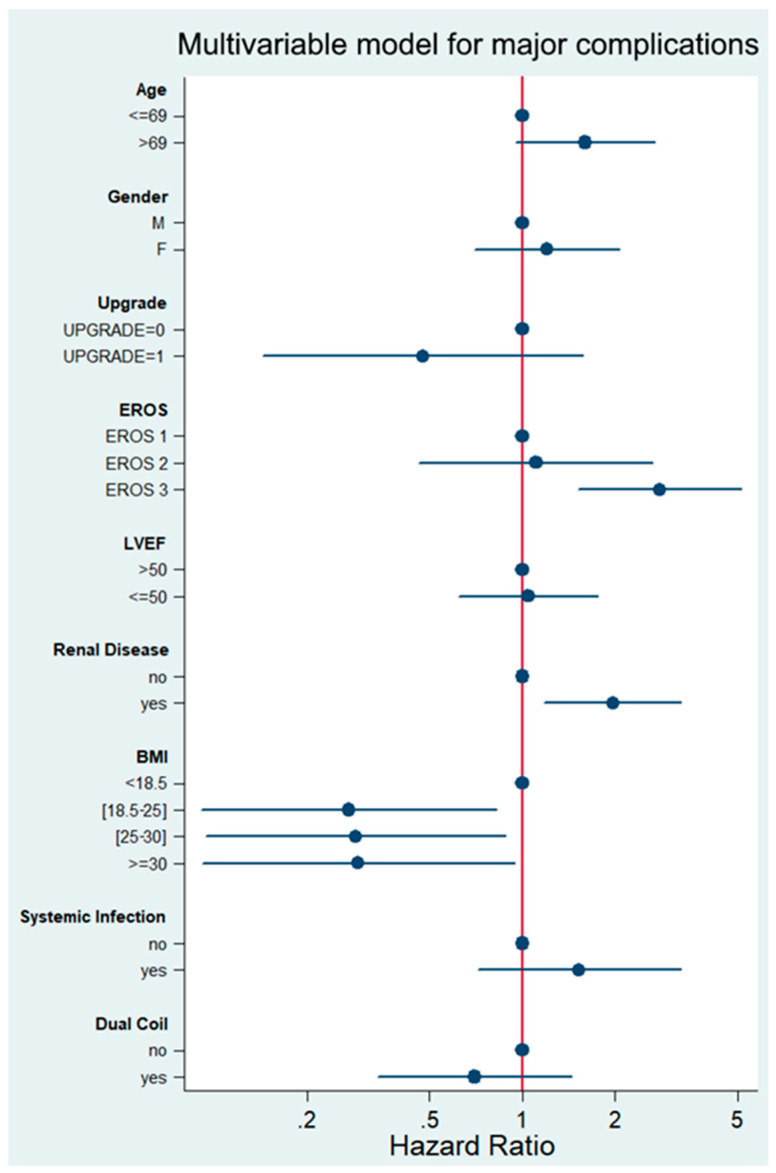
Multivariable analysis of predefined variables for major complications after TLE. Hazard ratios for each variable are shown.

Multivariable analysis on the predefined variables was performed to identify factors associated with any major complication at 30 days. The full multivariable model (Hazard ratios for each predefined variable) is shown in Figure 2.

Upgrade procedures per se were not associated with a greater risk for complications (HR 0.48, 95%-CI 0.14–1.57, *p* = 0.224). However, EROS risk group 3 (HR 2.79, 95%-CI 1.52–5.13, *p* = 0.001) was associated with major complications following TLE at 30 days. The other factor independently associated with increased risk was renal impairment (HR 1.96, 95% confidence interval 1.17–3.28, *p* = 0.010). 

**Figure 3:**
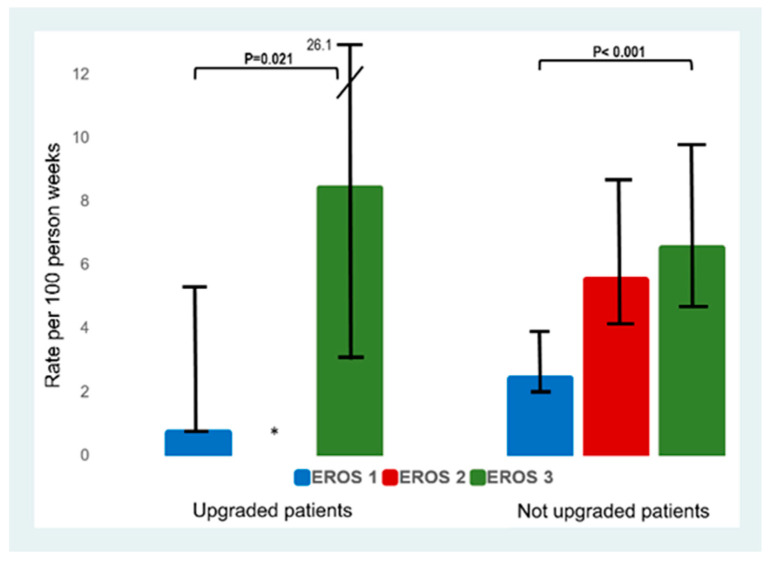
Rate of any complication according to EROS risk stratification for each group. * stands for no patients in this group.

As shown in Figure 3, upgraded patients in EROS 3 group (with long dwelling pacemaker (>15 years) or defibrillator leads (>10 years)), presented a significantly higher rate of major complications compared to upgraded patients classified in EROS 1 group. EROS 1 patients are patients with a shorter pacemaker (≤15 years) or defibrillator lead dwell time (≤10 years) and no congenital heart disease, implantation at a young age, infectious TLE indication, kidney disease, or chronic heart failure. There was also a statistical trend towards a lower number of overall complications in centers with a higher number of TLEs (Spearman rank correlation coefficient −0.64, *p* = 0.096).

### 3.3. Comparative Outcomes of Upgraded Patients and Patients with Other Indications for TLE

Details on TLE procedures and outcomes for patients, who underwent upgrade and patients with other TLE indications are shown in Table 2. Patients that were upgraded had—per the definition—more often a simple pace/sense electrode in place at the time of intervention (89.2% vs. 66.8%, *p* < 0.001). Dual-coil leads were less common in the upgraded group as well (*p* = 0.013). 

Rotational mechanical sheaths were more often used in upgraded patients (59% vs. 42.7%, *p* = 0.015). Device upgrades were associated with a longer total procedure time (160 min vs. 105 min, *p* < 0.001) and fluoroscopy duration (20.5 min vs. 10.6 min, *p* = 0.030). However, this was related to the re-implant procedure (extraction time was not different). Clinical success rates were not different between groups (97.6% vs. 93.0%, *p* = 0.569), as were radiological success rates (*p* = 0.351). Complete radiological success was achieved in 92.8% of upgraded patients and 88.1% of patients with other TLE indications (*p* = 0.351).

**Figure 4:**
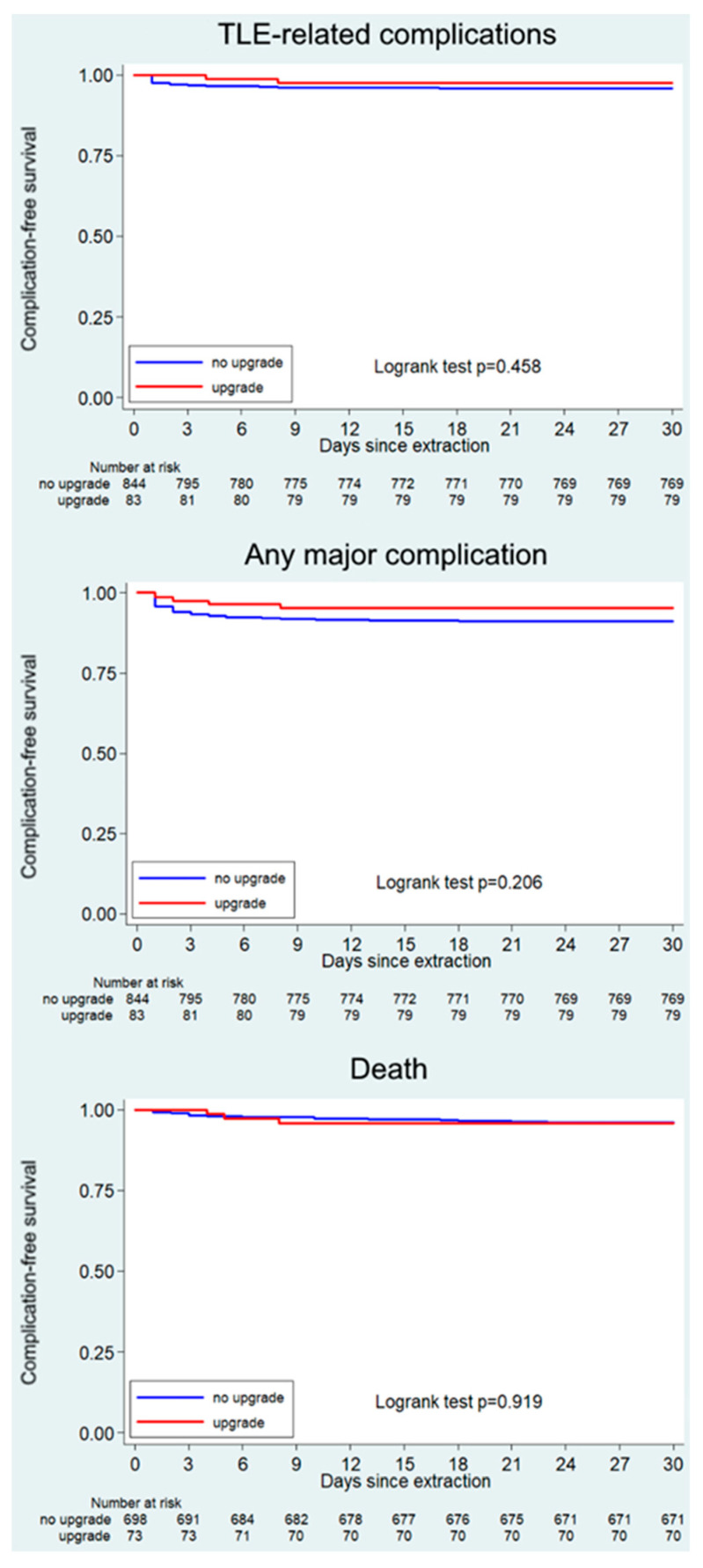
Comparison of TLE-related complications (**top**), any major complication (**middle**), and death (**bottom**) for cause-free survival between upgraded patients (red) and patients who underwent TLE due to other indications (blue).

The rate of acute complications, mortality and events in the postprocedural 30-day follow-up or postprocedural complications and mortality was not different (Figure 4). 

A detailed overview of the observed acute and post-procedural (30-day follow-up) complications is provided in Table 3. Acute major complications occurred in 7.2% of upgraded patients and 5.5% of patients with other TLE indications (*p* = 0.457). Intraprocedural complications of upgraded patients were mostly associated with the implantation and not the extraction procedure (67% vs. 33% of complications, respectively). 

## 4. Discussion

In this large registry study, we aimed to investigate patient and procedural factors influencing TLE outcomes in patients receiving a device upgrade. The main findings of this nationwide registry are:

1. The incidence of upgrade-associated TLE has increased by a factor of three in the last years compared to the number of such interventions almost ten years ago;
2. TLE success rates during device upgrade interventions were >97% despite a considerable median lead dwell time of >6.5 years;
3. In upgraded patients, the intraprocedural mortality rate was 2.4%, the rate of acute major complications was 7.2%, and an additional 1.2% of patients experienced complications during the first 30 days after TLE. Complication rates were not different than in other patients;
4. TLE during device upgrade in patients with long-dwelling pacemakers (>15 years) or defibrillation (>10 years) leads is associated with a higher risk of major complications at 30 days [15].

### 4.1. Efficacy of TLE during Device Upgrade

In this registry, we observed clinical TLE success rates in upgraded patients of 97.6%. This was numerically more (not statistically significant) compared to patients that had other TLE indications. These success rates were also slightly higher than reported outcome data from other larger and newer registries such as ELECTRa and PROMET [5,6]. These favorable success rates in upgraded patients compared to a general TLE cohort are in line with previous reports by Barakat et al. and Stefańczyk et al., who assessed a similar subset of patients undergoing device upgrade [12,13]. Regarding the high clinical success rate, it is important to note that not all leads are necessarily targeted during a TLE due to a planned upgrade and some of the intact leads may be re-used or—in case of especially difficult or risky extraction maneuvers—just be abandoned. Such a strategy, however, comes at the risk of the damage of coexisting leads, which may remain unnoticed during TLE. It is estimated that 4–32% of leads that are not primarily targeted will sustain inadvertent damage during TLE, eventually requiring extraction at a later stage [12,16]. 

### 4.2. Safety of TLE during Device Upgrade

We observed 7.2% acute major complications in upgraded patients, whereof 2.4% were related to the extraction and 4.8% to the re-implant/upgrade procedure (e.g., lead dislocation). TLE-associated major complications were reported to be in the range of 1–2.1% in general TLE patients [5,6,7,17,18], which is slightly less than in our study. For the specific subset of upgraded patients, complication rates of ~1% have been reported [12]. At the time of the interventions, most Swiss centers in our registry were low-volume centers according to established criteria (annual TLE rates of <30 procedures/year) [14], which might have contributed to the observed rate of complications. Several large previous studies have reported a higher complication rate in low-volume centers [5,7,15,19], with an odds ratio for complications of almost two [15]. This is partially also attributable to operators with limited experience [20,21]. Indeed, from a sub-analysis of the Swiss TLE registry, it was shown that outcomes can improve over time with the transformation into a high-volume center [22].

### 4.3. Study Limitations

The present study is a retrospective analysis of data from the nationwide Swiss Lead Extraction registry comparing early outcomes after TLE. The generalizability of our results to other regions and centers may be limited and depend on—amongst other factors—the extraction policy of contributing centers, TLE indications, the procedural setting, operator experience, and available technologies/resources. As this is a post hoc analysis, selection and detection bias may not be ruled out completely, although the data have been retrieved carefully from the hospital’s health records. Some variables (e.g., fluoroscopy time) suffered from a significant amount of missing values. Moreover, some differences in the baseline characteristics of the two patient groups and unknown co-variates may have influenced the comparative analysis.

## 5. Conclusions

TLE during device upgrade is successful most of the time and does not add a disproportionate risk to device upgrade procedures. However, patients with a longer dwelling time of pacemaker or defibrillation leads are at higher risk of having major complications. Risks and benefits of TLE in such patients should carefully be weighed, especially when other known risk factors are present, such as renal impairment or low body mass index. 

## Figures and Tables

**Table 1:** Patient baseline characteristics.

Variable	Upgrade Indication (n = 83)	Other Indications (n = 858)	Missing Data (%)	*p*-Value
Age	65.5 (14.1)	65.7 (15.9)	1.3%	0.668
Male gender	68 (81.9%)	601 (70.0)	1.3%	0.031
Body mass index [kg/m^2^]	27.4 (5.4)	26.6 (5.1)	6.1%	0.363
Hypertension	44 (53.0%)	444 (51.7%)	2.9%	1.000
Diabetes mellitus	14 (16.9%)	177 (20.6%)	3.2%	0.475
Dyslipidemia	38 (45.8%)	389 (45.3%)	3.2%	1.000
Chronic kidney disease	18 (21.7%)	216 (25.2%)	5.4%	0.429
Chronic obstructive pulmonary disease	7 (8.4%)	73 (8.5%)	6.2%	0.834
Known heart disease			6.3%	0.038
Coronary artery disease (CAD)	28 (33.7%)	262 (29.4%)		
Hypertrophic cardiomyopathy	2 (2.4%)	29 (3.4%)		
Arrhythmogenic dysplasia	1 (1.2%)	13 (1.5%)		
Channelopathy	0 (0.0%)	11 (1.3%)		
None	9 (10.8%)	163 (19.0%)		
Other heart disease	43 (51.8%)	380 (44.3%)		
Left ventricular ejection fraction (%)	36.4 (11.9)	47.2 (15.0)	6.5%	<0.001
Medication				
Anticoagulation	50 (60.2%)	403 (47.0%)	3.1%	0.050
Anti-platelet therapy	29 (34.9%)	288 (33.6%)	3.1%	1.000
Digoxin	2 (2.5%)	23 (2.7%)	4.6%	1.000
Diuretics	55 (66.3%)	431 (50.2%)	3.9%	0.020
ACEI/ARB/ARNI	60 (72.3%)	492 (57.3%)	4.1%	0.023
Beta-blocker	62 (74.7%)	526 (61.3%)	3.5%	0.053
Calcium-antagonist	12 (14.5%)	89 (10.4%)	4.4%	0.357
Anti-arrhythmic drugs	9 (10.8%)	127 (14.8%)	3.9%	0.333
Anti-aldosteronic agent	26 (31.3%)	185 (21.6%)	3.9%	0.077
Statin	38 (45.8%)	388 (45.2%)	3.9%	0.818
Implanted device history			1.5%	<0.001
Single/dual chamber pacemaker	62 (74.7%)	389 (45.3%)		
Single/dual chamber ICD	12 (14.5%)	257 (30.0%)		
CRT-P	7 (8.4%)	28 (3.3%)		
CRT-D	2 (2.4%)	170 (19.8%)		
Indication for extraction			1.3%	<0.001
Device upgrade	83 (100.0%)	0 (0.0%)		
Lead malfunction	0 (0.0%)	426 (49.7%)		
Infection	0 (0.0%)	337 (39.3%)		
Venous stenosis or occlusion	0 (0.0%)	11 (1.3%)		
Chronic pain	0 (0.0%)	10 (1.2%)		
Recalled lead	0 (0.0%)	7 (0.8%)		
Other	0 (0.0%)	67 (7.8%)		

**Table 2:** Procedural details and outcomes.

Variable	Upgrade Indication (n = 83)	Other Indications (n = 858)	Missing Data (%)	*p*-Value
TLE approach			2.4%	0.110
Superior left	56 (67.5%)	612 (71.3%)		
Superior right	18 (21.7%)	180 (21.0%)		
Superior left and right	6 (7.2%)	32 (3.7%)		
Superior left and femoral/jugular	0 (0.0%)	4 (0.5%)		
Superior right and femoral/jugular	3 (3.6%)	6 (0.7%)		
Femoral only	0 (0.0%)	1 (0.1%)		
Lead targeted for TLE			-	
Pacing and sensing lead	132 (89.2%)	1020 (66.8%)		<0.001
ICD single coil	7 (4.7%)	254 (16.6%)		0.047
ICD dual coil	6 (4.1%)	158 (10.4%)		0.013
CS bipolar	2 (1.4%)	43 (2.8%)		0.425
CS multipolar	1 (0.7%)	39 (2.6%)		0.248
Number of targeted leads per patient	1.7 [1–2]	1.8 [1–2]		1.000
Median lead dwelling time	6.6 [3.1–11.9]	6.2 [3.0–9.8]		0.072
TLE technique			3.9%	
Rotational mechanical	49 (59.0%)	366 (42.7%)		0.015
Laser sheath	11 (13.3%)	214 (24.9%)		0.011
Mechanical non-powered	12 (14.5%)	135 (15.7%)		0.755
Stylet only (manual traction)	8 (9.6%)	68 (7.9%)		0.677
Surgical	0 (0.0%)	1 (0.1%)		1.000
Other tools	3 (3.6%)	37 (4.3%)		1.000
Procedural data				
Total extraction time [min]	18 [15–25]	15 [10–25]	39.3%	0.674
Total procedure time [min]	160 [100–195]	105 [65–150]	16.3%	<0.001
Total fluoroscopic time [min]	20.5 [7.9–34.0]	10.6 [5.0–24.0]	49.9%	0.030
Radiological and clinical success				
Radiological success			2.9%	0.351
Complete	77 (92.8%)	756 (88.1%)		
Failure	0 (0.0%)	24 (2.8%)		
Partial	5 (6.0%)	52 (6.1%)		
Clinical success	81 (97.6%)	798 (93.0%)		0.569

**Table 3:** Acute and postprocedural complications.

Variable	Upgrade Indication (n = 83)	Other Indications (n = 858)	Missing Data (%)	*p*-Value
Acute major complications	6 (7.2%)	47 (5.5%)	-	0.457
Cardiac avulsion or tear	0 (0.0%)	4 (0.5%)		
Vascular avulsion or tear	0 (0.0%)	6 (0.7%)		
Major thromboembolic event	0 (0.0%)	3 (0.3%)		
Pneumothorax	0 (0.0%)	2 (0.2%)		
Stroke/TIA	1 (1.2%)	0 (0.0%)		
Complication associated with re-implant	4 (4.8%)	21 (2.4%)		
Other	1 (1.2%)	11 (1.3%)		
Intraprocedural TLE-related death	2 (2.4%)	11 (1.3%)	-	0.321
Postprocedural complications	1 (1.2%)	33 (3.8%)	1.4%	0.354
Death (not procedure-related)	1 (1.2%)	6 (0.7%)		
Heart failure	0 (0.0%)	4 (0.5%)		
Pocket hematoma	0 (0.0%)	6 (0.7%)		
Progressive renal failure	0 (0.0%)	3 (0.3%)		
Pneumonia	0 (0.0%)	1 (0.1%)		
Recurrence of infection/sepsis	0 (0.0%)	4 (0.5%)		
Other	0 (0.0%)	9 (1.0%)		

## Data Availability

Data are available upon request from the author’s consortium.
